# Seasonal Variation in Viral Infection Rates and Cell Sizes of Infected Prokaryotes in a Large and Deep Freshwater Lake (Lake Biwa, Japan)

**DOI:** 10.3389/fmicb.2021.624980

**Published:** 2021-05-11

**Authors:** Shang Shen, Yoshihisa Shimizu

**Affiliations:** Research Center for Environmental Quality Management, Kyoto University, Kyoto, Japan

**Keywords:** viral lysis, freshwater lake, transmission electron microscopy, seasonal variation, prokaryotic mortality, cell volume

## Abstract

As viruses regulate prokaryotic abundance and the carbon cycle by infecting and lysing their prokaryotic hosts, the volume of infected prokaryotes is an important parameter for understanding the impact of viruses on aquatic environments. However, literature regarding the seasonal and spatial variations in the cell volume of infected prokaryotes is limited, despite the volume of the prokaryotic community varying dynamically with season and water column depth. Here, we conducted a field survey for two annual cycles in a large and deep freshwater lake (Lake Biwa, Japan), where large prokaryotes inhabit the deeper layer during the stratified period. We used transmission electron microscopy to reveal the seasonal and spatial variation in the frequency of viral infection and cell volume of infected prokaryotes. We found that the viral infection rate in the surface layer increased when estimated contact rates increased during the middle of the stratified period, whereas the infection rate in the deeper layer increased despite low estimated contact rates during the end of the stratified period. In addition, in the deeper layer, the fraction of large prokaryotes in the total and infected prokaryotic communities increased progressively while the number of intracellular viral particles increased. We suggest different ways in which the viral abundance is maintained in the two water layers. In the surface layer, it is speculated that viral abundance is supported by the high viral infection rate because of the high activity of prokaryotes, whereas in the deeper layer, it might be supported by the larger number of intracellular viral particles released from large prokaryotes. Moreover, large prokaryotes could contribute as important sources of organic substrates *via* viral lysis in the deeper layer, where labile dissolved organic matter is depleted.

## Introduction

Since the discovery that prokaryotes-infecting viruses (bacteriophages) are numerically the most abundant entities in aquatic environments, there has been significant progress in research on viral ecology in aquatic environments, especially over the last three decades. Infection of prokaryotes by viruses leads to prokaryotic cell lysis and channels the prokaryotic biomass to the pool of dissolved organic matter (DOM), thus hampering protist grazing and the flow of nutrients in prokaryotes to higher trophic levels (Suttle, 2005). Along with the viral infection, grazing by protists is a major contributor to prokaryotic mortality (Fuhrman and Noble, 1995). On average, 10–60% (up to 100%) of the prokaryotes produced daily are killed by viral infections (Weinbauer and Höfle, 1998a; Brum et al., 2005; Pradeep Ram et al., 2010b). In addition, virus-released lysates are highly bioavailable and are quickly utilized by prokaryotes (Middelboe and Jørgensen, 2006; Zhao et al., 2019), suggesting that viral infection and prokaryotic cell lysis enhance nutrient cycling in aquatic systems. Thus, viruses not only regulate the abundance and diversity of their hosts but also contribute to the carbon and nutrient fluxes of their environments.

To quantify seasonal and spatial variations in virus-produced lysates, one needs to consider not only the frequency of infection and prokaryotic productivity but also the volume of infected cells. Recently, the predominance of a prokaryotic group with large cell sizes (clade CL500-11, phylum Chloroflexi, cell length: 1–2 μm) was reported in the deeper layers of various oxygenated deep freshwater lakes (Denef et al., 2016; Okazaki et al., 2017, 2018). For instance, in Lake Biwa (Japan), CL500-11 is predominant (up to 25.9%) in the deeper layer (50–70 m) during the stratified period (Okazaki et al., 2013, 2017). During this period, the median cell volume of the prokaryotic community in the lake is up to 7-fold greater in the deeper layer than in the surface layer (Shen and Shimizu, 2020). Differences in the volumes of virus-infected cells between deeper and surface layers (or stratified and de-stratified periods) would directly influence the amount of cell lysate produced after virus-induced lysis. However, very little is known about seasonal variations in the volumes of infected cells in deep freshwater lakes (Brum et al., 2005) or the relationship between the infected and total cell volumes of the prokaryotic communities (Weinbauer and Höfle, 1998b). To the best of our knowledge, no previous study has reported on these relationships in deep freshwater lakes, where large prokaryotes are reported to predominate. If the proportion of infected prokaryotes with large cell sizes increases in the deeper layer during the stratified period, when the proportion of large prokaryotes increases, the impact of viral infection could be significant. This is because virus-induced lysates are rapidly consumed by other prokaryotes (Middelboe and Jørgensen, 2006) and are an important source of consumable organic matter in the deeper layer, where the supply of new organic carbon (e.g., through primary production) is limited. Thus, analyzing the cell volumes of infected prokaryotes is important to estimate the impact of viral infection on carbon cycling.

In this study, we examined prokaryotic mortality in response to viral infection and lysis, in addition to the size distribution of infected prokaryotic cells during different seasons at the surface of Lake Biwa and in its deeper layers. Our goal was to reveal the relationship between the cell volumes of entire prokaryotic communities and those of infected cells in Lake Biwa, where the proportion of large prokaryotes is increased in the deeper layer.

## Materials and Methods

### Sampling Site and Sample Collection

Lake Biwa, located in the west-central Honshu Island, is the largest deep (maximum depth; 104 m), monomictic freshwater lake in Japan (surface area; 674 km^2^). Lake water samples (500 ml) were collected monthly from the surface (depth: 0.5 m) and the deeper layer (depth: 60 m) using sterile bottles and a Van Dorn water sampler at the pelagic station in Lake Biwa (35° 23' 41''N, 136° 07' 57''E) from August 2016 to January 2018 (18 months, 36 samples in total). Buffered glutaraldehyde (pH 7.2, final concentration: 1%) was used to immediately fix the samples collected on board. Fixed samples were incubated at 4°C prior to transportation to the laboratory and stored at −30°C until further analysis.

### Enumeration of Viral Abundance

To assess viral abundance, the DNA staining method was used (Shibata et al., 2006; Patel et al., 2007). Briefly, fixed subsamples (0.5–2 ml) were filtered using Anodisc filters (pore size: 0.02 μm, Whatman), and a 0.8-μm-pore size filter (mixed cellulose esters membrane filters) was used as a backing filter. Anodisc filters were air-dried and stained using SYBR Gold (Molecular Probes; stock solution diluted 1:400). After staining, the filters were air-dried again, and each filter was mounted between a slide glass and a coverslip using anti-fade mounting medium [0.1% (vol/vol) *p*-phenylenediamine]. Slides were kept at −30°C prior to counting. The slides were observed under an epifluorescence microscope (BZ-9000, KEYENCE) at 1,000× magnification. Ten fields and at least 200 virus-like particles (VLPs) were counted per sample. A blank was constructed with the same method but using ultrapure water to check for contamination from reagents or equipment.

### Frequency of Visibly Infected Cells and Number of Intracellular Viral Particles

For evaluating prokaryotic mortality due to viral lysis, the FVIC was estimated using transmission electron microscopy (TEM, H-7650, Hitachi), following a previous study (Pradeep Ram et al., 2010b) except that EM stain (Nisshin EM Co. Ltd.) was used instead of uranyl acetate – the use of which is severely regulated – to electronically stain prokaryotic cells. The fixed lake water samples were prefiltered through 10-μm-pore size polycarbonate filters to remove large particles. Prokaryotic cells in the fixed samples were collected onto TEM grids (Cu-400-mesh, carbon-coated Formvar film) by ultracentrifugation (Himac CS 100GXII, Hitachi; S52ST Swing-Out-Rotor at 70,000 × *g* for 20 min at 4°C); triplicate grids were prepared for each sample. Each grid was stained at room temperature (25°C) for 30 min using EM stain (positive staining; 1.0-fold; Shen et al., 2017). The stained grids were rinsed with ultrapure water five times to remove excess staining solution and then air-dried overnight. The samples were observed under TEM operated at 80 kV with magnifications of 3,000–50,000. More than 500 prokaryotic cells were examined per grid to determine the FVIC. A prokaryotic cell was considered as infected when three or more viruses were clearly observed inside it. FVIC was converted to frequency of infected cells (FIC) using the empirically equation (Weinbauer et al., 2002): FIC = 9.524 FVIC − 3.256, considering that only the mature viruses are visible upon using TEM. FIC was converted to fraction of prokaryotic mortality due to viral lysis (FMVL) using the equation based on a theoretical model (Binder, 1999): FMVL = (FIC + 0.6 FIC^2^)/(1–1.2 FIC). The number of intracellular viral particles was estimated by counting the number of viruses, which were clearly visible inside an infected cell. Sizes of the infected cells were calculated by assuming that the cell shape was cylindrical with a hemisphere at each end. Prokaryotic morphotypes were operationally classified using length (L) and width (W) of cells as follows (Racy et al., 2005): cocci (1 ≤ L/W < 1.25), coccobacilli (1.25 ≤ L/W < 1.75), short rods (1.75 ≤ L/W < 5), and elongated rods/filaments (5 ≤ L/W).

### Estimation of Nutrient-Release Rate by Viral Lysis

Carbon and nitrogen release rates (μg L^−1^ d^−1^) due to viral lysis were estimated by multiplying prokaryotic production, prokaryotic biomass, and FMVL (%). Datasets of prokaryotic production were obtained from a previous study, which was conducted in Lake Biwa (a pelagic site) during the same month and year (Tsuchiya et al., 2020). Datasets of prokaryotic biomass were obtained from a previous study, which was conducted in Lake Biwa at the same sampling station and on the same date (Shen and Shimizu, 2020). Values for prokaryotic cell carbon and nitrogen contents (106 fg of C μm^−3^ and 25 fg of N μm^−3^) were referenced to published literature (Nagata, 1986). The release of phosphorus due to viral lysis was calculated based on the datum that the P/C ratio is 0.04 (Cole et al., 1993).

### Virus-Host Contact Rate

To estimate the contact rate between viruses and prokaryotes (contacts d^−1^) every month at each depth, the following equation was used (Murray and Jackson, 1992):

$$R=\left(2\pi dD_{v}S\right)VB$$

where *d* is the prokaryotic cell length (median, cm; Shen and Shimizu, 2020), *D_v_* is the diffusivity of viruses, *V* is the viral abundance (VLPs ml^−1^), *B* is the prokaryotic abundance (cells ml^−1^; Shen and Shimizu, 2020), and *S* is the Sherwood number (dimensionless). *D_v_* was calculated from *D_v_* = *kT/3πμdv*, where *k* is the Boltzmann constant (1.38 × 10^−23^ J K^−1^), *T* is the water temperature (K), *μ* is the viscosity of water (pascals per s, at *in situ* water temperature), and *dv* is the diameter of the viral capsid (median value = 98.5 in Lake Biwa, N = 22). The Sherwood number of prokaryotic communities has previously been determined as 1.06 (Wilhelm et al., 1998), assuming that 10% of prokaryotes in communities are motile (*S* = 1.53) while 90% are nonmotile (*S* = 1.01; Mitchell et al., 1995). The number of daily viral contacts per prokaryotic cell every month at each depth (contacts cell^−1^ d^−1^) was calculated by dividing the contact rate by the prokaryotic abundance.

### Bulk Dissolved Organic Carbon Analysis

The collected water samples were filtered through a pre-combusted (450°C for 4 h) and prewashed GF/B filter (Whatman). DOC concentrations in the filtrates were measured as non-purgeable organic carbon using a high-temperature combustion analyzer (TOC-V_CPH_, Shimadzu) equipped with a CO_2_ detector.

### Data Analyses

Statistical analyses were performed using the R software (ver. 3.5.1; R Core Team, 2018), and the figures were prepared using the ggplot2 package (Wickham, 2016). Student’s *t*-test was applied to test the statistical significance of observed differences in the abundance and FVIC and when values met normality but not equal variance assumption, Welch’s *t*-test was applied. For abundance and FVIC, each sample (each month) or pooled samples (stratified and de-stratified periods) were compared (Supplementary Table 1). As some samples did not meet normality, the Mann-Whitney *U* test was applied to test the statistical significance of observed differences in the cell volume distributions of different samples pooled by depths or seasons (see Determination of Stratified and De-stratified Periods section for detail). A value of *p* less than 0.05 was considered as statistically significant.

## Results

### Determination of Stratified and De-stratified Periods

**Figure 1:**
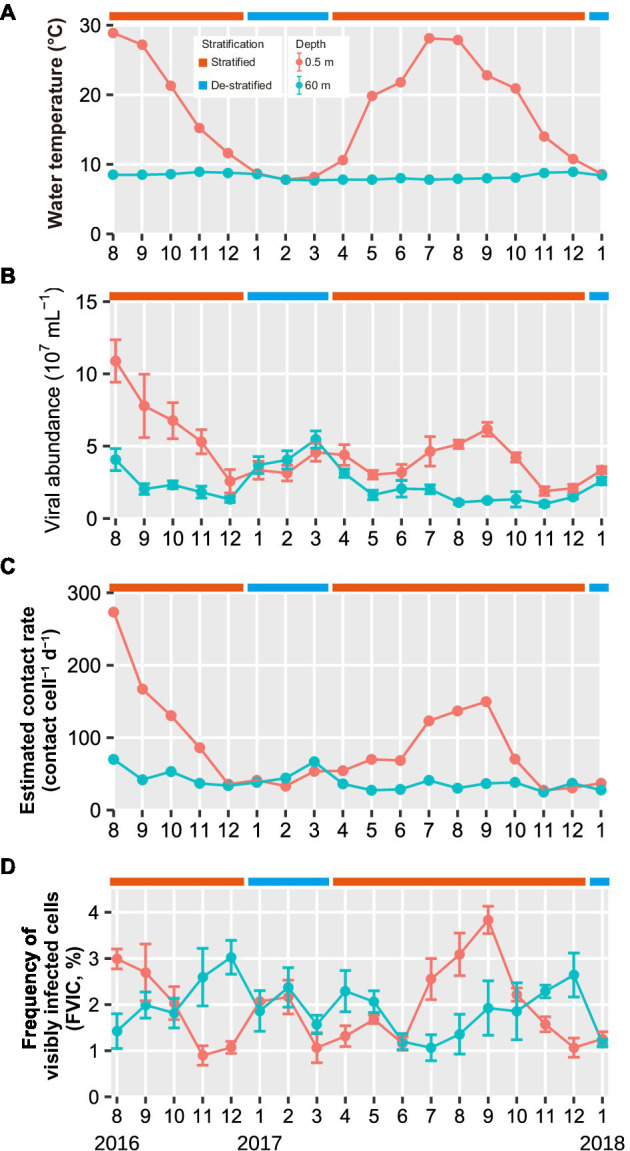
Seasonal variations in **(A)** water temperature **(B)** viral abundance, **(C)** estimated contact rates, and **(D)** FVIC in the surface and deeper layers throughout the study period (August 2016 to January 2018). Months are represented as 1–12. Values in panel (**B**,**D**) shown are the mean ± standard deviation (*n* = 10 and 3 in (**B**,**D**), respectively). The water temperature data were obtained from Shiga-Prefecture environmental white paper (Shiga-Prefecture, 2017, 2018). Results of statistical analyses of viral abundance and FVIC are shown in Supplementary Table 2.

In this study, two periods, a period of stratification and a period of vertical mixing (destratification) within the water column, were determined from datasets of the vertical profiling (at depths of 0–90 m) of the water temperature and dissolved oxygen concentration (Shiga-Prefecture, 2017, 2018). We defined the de-stratified periods as the times when the concentrations of dissolved oxygen (DO) were same in the surface and deeper layers and difference in the water temperatures between the layers was <1°C. The stratified periods, during which water temperatures ranged from 10.8 to 28.1°C in the surface layer and 7.8 to 8.9°C in the deeper layer, occurred from August to December in 2016 and April to December in 2017 (Figure 1A). Moreover, we divided the stratified period into three periods: beginning (April–June), middle (July–September), and end (October–December). The de-stratified periods, during which the water temperatures in the absence of a thermocline ranged from 7.7 to 8.7°C, occurred from January to March 2017 and during January 2018.

### Viral Abundance and Contact Rate

During the stratified periods, viral abundance in the surface layer was higher (Welch’s *t*-test and Student’s *t*-test, *p* < 0.05) and more variable (1.9–11 × 10^7^ VLPs ml^−1^) than that in the deeper layer (0.97–4.1 × 10^7^ VLPs ml^−1^; Figure 1B; Supplementary Table 2). During the de-stratified period, both layers exhibited similar viral abundance (Welch’s *t*-test and Student’s *t*-test, *p* > 0.05), which ranged from 2.9 × 10^7^ to 6.0 × 10^7^ VLPs ml^−1^ in the surface layer and 2.6 × 10^7^ to 5.1 × 10^7^ VLPs ml^−1^ in the deeper layer. During the stratified period, the viral contact rate varied from 27 to 273 contacts cell^−1^ d^−1^ in the surface layer, whereas the value in the deeper layer was less variable and ranged from 25 to 70 contacts cell^−1^ d^−1^ (Figure 1C; Table 1).

**Table 1:** Statistical significance in the cell volume differences between the surface and deeper layers assessed using Mann-Whitney *U* test.

	Surface vs. deeper layer			
	De-stratified	Stratified-beginning	Stratified-middle	Stratified-end
Total prokaryotic community	(0.12)^NS^	(6.7 × 10^−5^)^***^	(1.7 × 10^−50^)^***^	(3.4 × 10^−58^)^***^
Infected prokaryotes	(0.90)^NS^	(0.77)^NS^	(8.9 × 10^−7^)^***^	(1.8 × 10^−10^)^***^

The value in each cell indicates the p-value. Original data is shown in Figure 3. *U*-values are shown in Supplementary Table 3. The median cell volumes were higher in the deeper layer for all comparisons that indicated significant differences.

* *p* < 0.05;

** *p* < 0.01;

*** *p* < 0.001.

NS No significant difference.

### The FVIC Depends on Stratification and the Annual Cycle

FVIC in the surface layer ranged from 0.9 to 3.8%; it exhibited an annual cycle, wherein surface layer FVIC tended to increase in summer (3.0% in August 2016 and 3.8% in September 2017) and decrease at the end of the stratified period (0.9% in November 2016 and 1.1% in December 2017; Figure 1D). FVIC in the deeper layer ranged from 1.1 to 3.0%; its values also followed an annual cycle, increasing at the end of the stratified period (3.0% in December 2016 and 2.6% in December 2017) and subsequently decreasing following lake turnover and re-stratification (1.1% in July 2017). During the de-stratified periods (i.e., January to March 2017 and January 2018), FVIC values were not significantly different between the surface and deeper layers (Student’s *t*-test, *p* > 0.05, Supplementary Table 2). FIC values were 6.9–33.3% and FMVL values were 8.0–66.9% during the study period.

### Size Distributions of Virus-Infected Cells Are More Variable in the Deeper Layer

**Figure 2:**
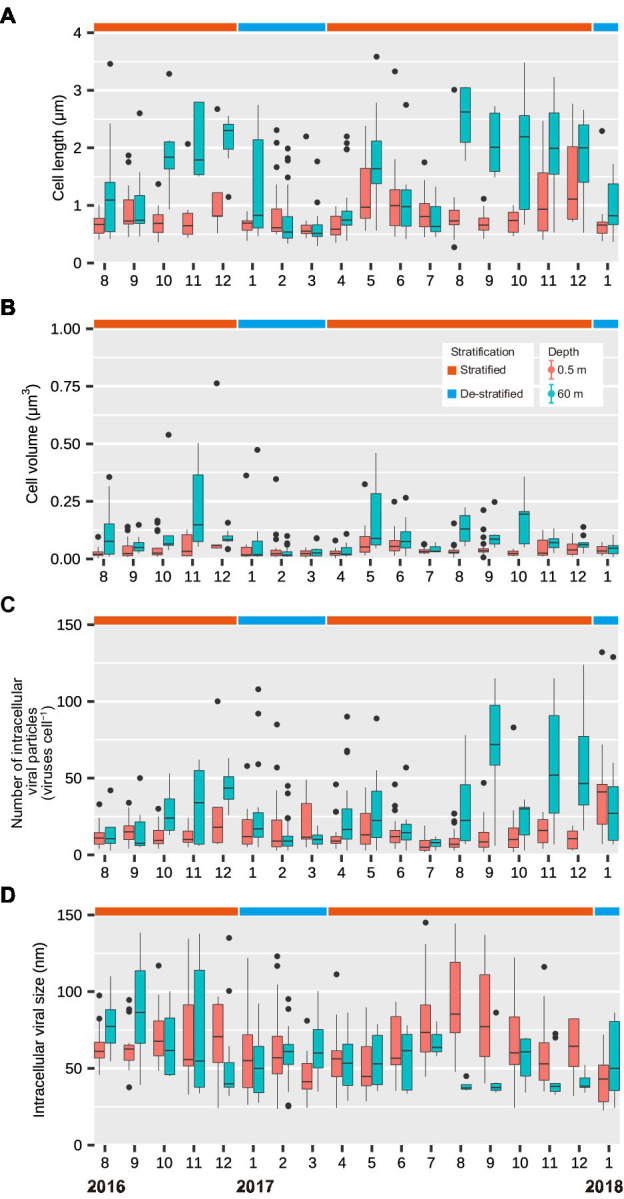
Seasonal variations in **(A)** the lengths of infected cells (μm), **(B)** the volumes of infected cells (μm3), **(C)** the number of intracellular viral particles (viruses cell−1), and **(D)** the intracellular viral capsid size (nm) in the surface and deeper layers throughout this study period (August 2016 to January 2018). Months are represented as 1–12. The top and bottom of each box show the 25th and 75th percentiles (Q1 and Q3), respectively. The interquartile range (IQR) = Q3 − Q1. The whiskers range from Q1–1.5 × IQR to Q3 + 1.5 × IQR. The middle line in each box indicates the median.

**Figure 3:**
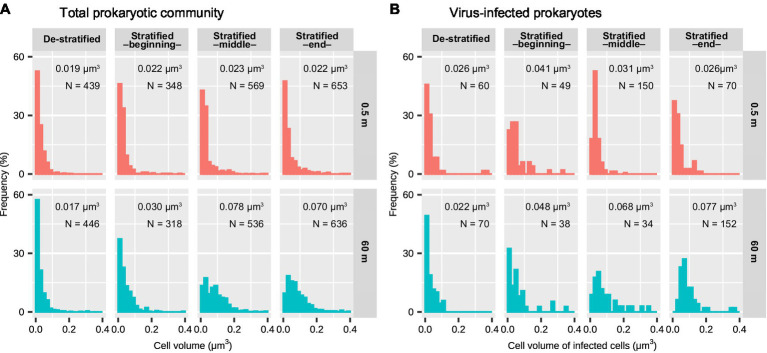
Cell volumes of **(A)** the total prokaryotic community and **(B)** virus-infected prokaryotes in the surface and deeper layer of Lake Biwa during the stratified and de-stratified periods. The width of each bar indicates 0.02 μm^3^. The values in each plot indicate median cell volume. **(A)** was adapted from a previous study (Shen and Shimizu, 2020). Results of the statistical analyses are shown in Tables 1 and 2.

During the stratified period, the lengths of infected cells ranged from 0.27 to 3.33 μm (median: 0.74 μm) in the surface layer and from 0.39 to 3.59 μm (median: 1.52 μm) in the deeper layer (Figure 2A). The cell volume of infected cells ranged from 0.0018 to 0.76 μm^3^ (median: 0.031 μm^3^) in the surface layer and from 0.010 to 0.54 μm^3^ (median: 0.065 μm^3^) in the deeper layer (Figure 2B). Lengths and volumes of infected cells in the deeper layer increased at the end of the stratified period and were higher than those in the surface layer (Mann-Whitney *U* test, *p* < 0.001, Table 1). During the de-stratified periods, the length and volume of virus-infected cells ranged from 0.30 to 4.34 (median: 0.63 μm) and 0.0061 to 1.26 μm^3^ (median: 0.024 μm^3^), respectively. Figure 3 shows histograms of the cell volumes of the entire prokaryotic community (adapted from Figure 2B in Shen and Shimizu, 2020) and of infected prokaryotes.

When comparing the surface layer with the deeper layer, both cell volume distribution of the total prokaryotic community and infected prokaryotes were not significantly different during the de-stratified period (Figure 3; Table 1). During the middle and end of the stratified period, both cell volume distribution of total prokaryotic community and infected prokaryotes in the deeper layer were significantly larger than those in the surface layer (Mann-Whitney *U* test, *p* < 0.001). In the surface layer, during the beginning and middle of the stratified period, cell volume distribution of infected prokaryotes was slightly but significantly larger than that of the total prokaryote community (Mann-Whitney *U* test, *p* < 0.001, Figure 3; Table 2). In the deeper layer, the cell volume distribution of infected prokaryotes was not significantly different, but was slightly larger than that of the total prokaryotic community through the stratified period.

**Table 2:** Statistical significance in the cell volume differences between the total prokaryotic community and infected prokaryotes assessed using Mann-Whitney *U* test.

	Total prokaryotic community vs. virus-infected prokaryotes			
	De-stratified	Stratified-beginning	Stratified-middle	Stratified-end
0.5 m	(0.16)^NS^	(7.3 × 10^−5^)^***^	(1.3 × 10^−4^)^***^	(0.17)^NS^
60 m	(0.039)^*^	(0.092)^NS^	(0.99)^NS^	(0.097)^NS^

The value in each cell indicates the value of *p*. Original data are shown in Figure 3. *U*-values are shown in Supplementary Table 4. The median cell volumes were higher for virus-infected prokaryotes for all comparisons that indicated significant differences.

* *p* < 0.05;

** *p* < 0.01;

*** *p* < 0.001.

NS No significant difference.

**Figure 4:**
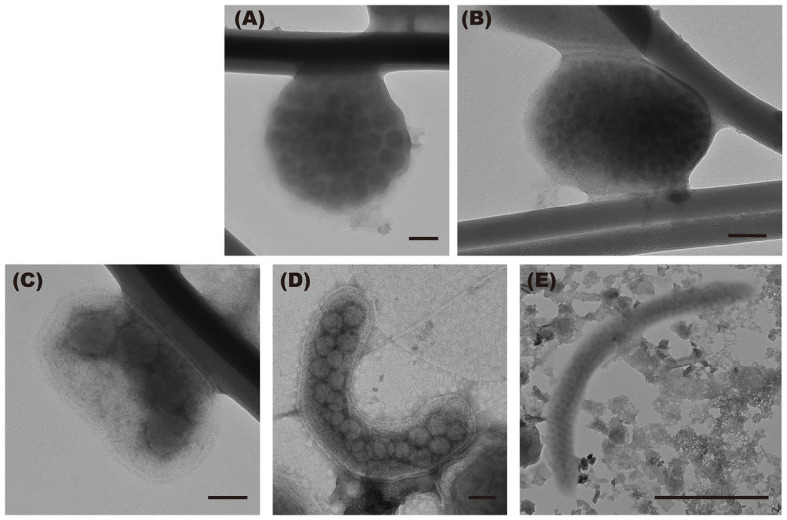
Transmission electron micrographs of virus-infected prokaryotic cells of the different morphology. **(A)** cocci, **(B)** coccobacilli, **(C)** short rods, and **(D,E)** elongated rods. Scale bar = 100 nm (500 nm in **E**).

**Figure 5:**
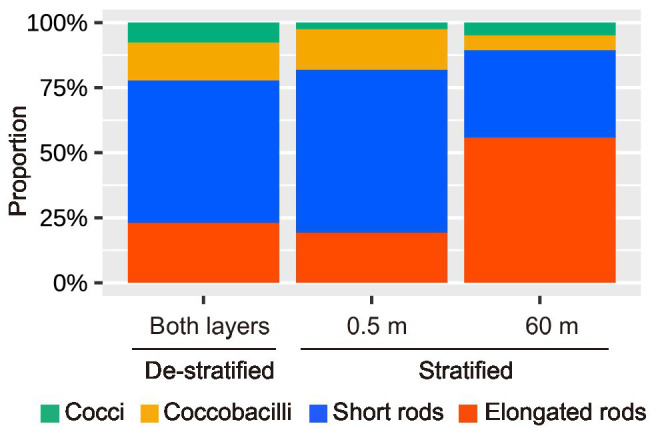
The proportion of the morphotypes of the infected prokaryotic cells during this study.

TEM images of virus-infected prokaryotic cells of different morphotypes are shown in Figure 4. Short rods were the major morphotype among virus-infected prokaryotes during the de-stratified period (54%) and in the surface layer during the stratified period (63%, Figure 5). In the deeper layer during the stratified period, most infected prokaryotes were elongated rods (56%).

### Number of Intracellular Viral Particles Increases as the Intracellular Viral Capsid Diameter Decreases

**Figure 6:**
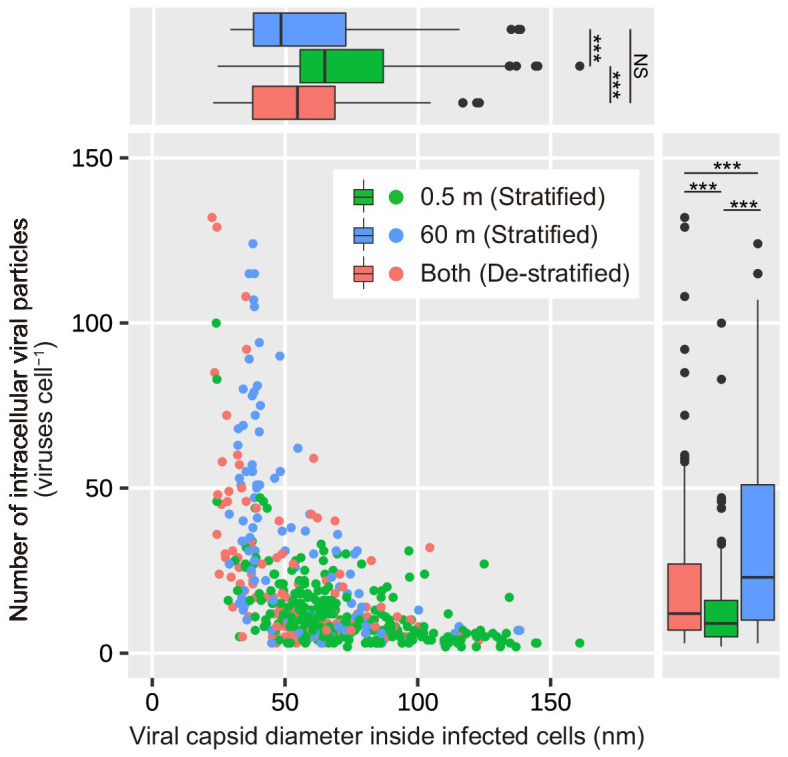
Dependence of the number of intracellular viral particles on the diameter of viral capsids inside the infected cells. The top and bottom of each box indicate the 25th and 75th percentiles (Q1 and Q3), respectively. The interquartile range (IQR) = Q3 − Q1. The whiskers range from Q1–1.5 × IQR to Q3 + 1.5 × IQR. The middle line in each box indicates the median. Results of the statistical analyses are shown in Supplementary Table 5. ^***^p < 0.001 and NS No significant difference.

During the stratified period, the number of intracellular viral particles in the surface layer varied from 3 to 100 viruses cell^−1^ (median: 9 viruses cell^−1^), whereas the intracellular viral capsid diameter in the surface layer ranged from 24 to 161 nm (median: 64 nm; Figures 2C,D; Table 1). In the deeper layer, the number of intracellular viral particles varied from 3 to 124 viruses cell^−1^ (median: 23 viruses cell^−1^), whereas intracellular viral capsid diameter varied from 29 to 139 nm (median: 48 nm). In the de-stratified periods, the number of intracellular viral particles varied from 3 to 132 viruses cell^−1^ (median: 12 viruses cell^−1^), and intracellular viral capsid diameter ranged from 22.5 to 123 nm (median: 54.3 nm). Figure 6 shows that the capsid diameters of intracellular viruses increased as the number of intracellular viral particles decreased.

### Relationship Between the Number of Intracellular Viral Particles and Cell Volume of Virus-Infected Prokaryotes

**Figure 7:**
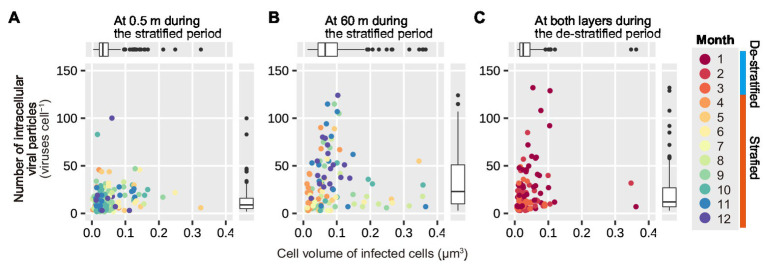
Correlation of the number of intracellular viral particles on the volume of infected cells. **(A,B)** show plots for the surface and deeper layers during the stratified period, respectively. **(C)** shows the plots for both the layers during the de-stratified period. The 12 colors indicate the 12 months. Each data point indicates a virus-infected prokaryote. Results of the statistical analyses are shown in Supplementary Table 5.

In the surface layer, during the stratified period, and in both the layers during the de-stratified period, data points were grouped mainly in the area with a small cell volume and small number of intracellular viral particles (in Figures 7A,C). However, in the deeper layer, during the stratified period, data points at the end of the stratified period (October–December) tended to be in the area with a larger number of intracellular viral particles and slightly large cell volume (in Figure 7B). Both cell volume and the number of intracellular viral particles in the deeper layer during the stratified period were significantly larger than those in the surface layer during the stratified period and those during the de-stratified period (Mann-Whitney *U* test, *p* < 0.001, Supplementary Table 5).

### Prokaryotic Mortality Due to Viral Lysis and Estimation of Nutrient-Release Rate

During the stratified periods, FMVL in the surface layer varied from 5.9 to 67% (median: 19%), corresponding to a range of 0.006 to 0.28 × 10^6^ cells ml^−1^ d^−1^. In the deeper layer, the values of FMVL varied from 7.9 to 43% (median: 21%), parallel to a range of 0.0018–0.0071 × 10^6^ cells ml^−1^ d^−1^. During the de-stratified periods, FMVL varied from 8.0 to 67% (median: 17%), consistent with a range of 0.0024–0.012 × 10^6^ cells ml^−1^ d^−1^.

During the stratified periods, the release rates of carbon, nitrogen, and phosphorus due to viral lysis were estimated to range from 0.018 to 0.89 μg C L^−1^ d^−1^, 0.043 to 0.21 μg N L^−1^ d^−1^, and 0.0007 to 0.036 μg P L^−1^ d^−1^, respectively, in the surface layer (Table 1). In the deeper layer, the C, N, and P release rates ranged from 0.013 to 0.057 μg C L^−1^ d^−1^, 0.0030 to 0.013 μg N L^−1^ d^−1^, and 0.0005 to 0.0023 μg P L^−1^ d^−1^, respectively. During the de-stratified periods, the values of the C, N, and P release rates ranged from 0.006 to 0.019 μg C L^−1^ d^−1^, 0.0014 to 0.0044 μg N L^−1^ d^−1^, and 0.0002 to 0.0008 μg P L^−1^ d^−1^, respectively.

### Bulk DOC Concentration

During the stratified periods, the concentrations of DOC varied between 1.1 and 1.4 mg C L^−1^ in the surface layer and between 0.94 and 1.1 mg C L^−1^ in the deeper layer (Table 1). During the de-stratified periods, concentrations of DOC ranged between 1.1 and 1.2 mg C L^−1^.

## Discussion

Our study aimed to reveal the relationship between viral lysis-induced prokaryotic mortality and infected cell size. We collected seasonal data on viral abundance, viral infection frequency, and the size of infected cells from the surface and deeper layers of Lake Biwa to accomplish our objective. These seasonal data yielded some interesting findings.

To discuss these results, we first need to outline the uncertainties inherent to the conversion models used to estimate FMVL. TEM is a powerful tool for the direct observation of viral infections. However, intracellular viruses can only be observed with TEM at the end of the viral life cycle, which means that TEM cannot detect intracellular viruses before the viral particles are assembled in a prokaryotic cell. Thus, an empirical model (FVIC to FIC) based on the viral dilution approach that considers the viral life cycle stages prior to viral particle assembly was proposed to calculate the real infection frequency (Weinbauer et al., 2002). A theoretical model with two important assumptions was employed to convert FIC to FMVL (Binder, 1999). First, prokaryotic production and mortality are in equilibrium in a water column. Second, grazers (protists) do not distinguish infected cells from uninfected cells when they consume prokaryotes. Parameters in this model depended on the individual aquatic system and were not optimized for Lake Biwa. Although we need to be cautious in our interpretation of the estimates of prokaryotic mortality resulting from viral lysis reported in this study, this approach has been used widely in recent publications (Pradeep Ram et al., 2010a,b), and our results are comparable with those of previous studies.

### Viral Abundance Is Maintained in Different Ways in Surface and Deeper Layers

The first important observation is that viral infection shows seasonal variation in both the surface and deeper layers. Infection rates increased in the summer in the surface layer (resulting in up to 66.9% of prokaryotic mortality), whereas in the deeper layer, infection rates increased in the end of the stratified period (resulting in up to 43.0% of prokaryotic mortality, Figure 1D). In the summer in Lake Biwa, prokaryotic production increased with increased water temperature in the surface layer, resulting in high prokaryotic abundance (Shen and Shimizu, 2020; Tsuchiya et al., 2020). This may cause high estimated contact rates (mean = 102 contacts cell^−1^ d^−1^), resulting in high infection rates. This suggests that although viral infection in the surface layer leads to a lower number of intracellular viral particles (one-third that in the deeper layer, Table 1), and viral abundance is supported by a high infection rate and high prokaryotic production. Compared to previous studies conducted in Lake Créteil (France) and Lake Vassivière (France), our result (mean FIC: 15.1%) was lower than that in Lake Créteil (FIC: mean, 32.0%) and similar to that in Lake Vassivière (FIC: mean, 17.6%; Pradeep Ram et al., 2010a, 2011). This could be explained in the same way. Lake Vassivière is mesotrophic like Lake Biwa, and these lakes had similar prokaryotic and viral abundances, resulting in similar estimated contact rates (174 contacts cell^−1^ d^−1^) and FIC values (Pradeep Ram et al., 2011). However, in Lake Créteil, prokaryotic and viral abundances were higher than in Lake Biwa, resulting in much higher estimated contact rates (549 contacts cell^−1^ d^−1^). Additionally, in another study, it has been reported that higher bacterial abundance and bacterial production in the surface microlayer compared to that in the underlying water maintains a higher viral abundance (Pradeep Ram et al., 2018).

However, at the end of the stratified period, the FVIC in the deeper layer was higher than that in the surface layer, despite lower contact rates between viruses and prokaryotes (mean = 38 contacts cell^−1^ d^−1^, Figures 1C,D). We speculate that there are three possible explanations for this. First, assuming that the majority of viruses in Lake Biwa infect only a single host species or a narrow range of species, and the diversity of prokaryotes and/or viruses is lower in the deeper layer than in the surface layer, the rate of productive contacts between prokaryotes and viruses may be increased, resulting in higher infection. However, viruses with broad host ranges have been reported in some studies (Waterbury and Valois, 1993; Lu et al., 2001), and reviewed in previous papers (Børsheim, 1993; Weinbauer, 2004). The second possibility is that these broad-spectrum viruses were increased in the deeper layer of Lake Biwa, resulting in more productive contacts. Broad host-range viruses are likely to be widespread when the abundance of hosts is low or variable (Sullivan et al., 2003; Elena et al., 2009). However, we did not examine the diversity of the prokaryotes or viruses, nor the viral host ranges. The last possibility is the existence of prophages, which switch from a lysogenic life cycle to a lytic one. This switching leads to capsid assembly in the host cells, resulting in a higher FVIC in the deeper layer. Viruses inhabiting the deep ocean likely undergo lysogeny due to the low activity of their hosts (Weinbauer et al., 2003; Luo et al., 2017). Lysogenic viruses are induced to enter the lytic cycle in response to changes in their environment (e.g., sunlight, organic matter, and nutrient concentrations). In Lake Biwa, N and P concentrations were increased in the deeper layer at the end of the stratified period (Shiga-Prefecture, 2016, 2017). This increase in nutrients may promote the activity of prokaryotes with large cell sizes and induce prophages to enter the lytic cycle. During the stratified period, the cell volumes of infected prokaryotes and their number of intracellular viral particles were higher in the deeper layer than in the surface layer (Figures 2B,C). The intracellular viral size was smaller in the deeper layer than in the surface layer (Figure 2D). Viral capsid diameter increased when the number of intracellular viral particles decreased (Figure 6). These results were consistent with previous studies, which reported that the number of intracellular viral particles increases with cell size and decreases with intracellular viral size (Weinbauer and Peduzzi, 1994; Weinbauer and Höfle, 1998b). When host cell activity is low, the latent period can be prolonged to increase the number of intracellular viral particles (Parada et al., 2006). It has also been reported that lysogenic viruses can have prolonged latent periods and large numbers of intracellular viral particles (Wilson and Mann, 1997).

### Larger Prokaryotes Are Associated With More Intracellular Viral Particles in the Deep Layer

In the surface layer, during the beginning and middle of the stratified period, the cell volume distribution of infected prokaryotes shifted to larger and significantly larger, respectively, compared to that of the total prokaryotic community (Figure 3; Table 2). Although the cell volume of the infected prokaryotes was not significantly different from that of the total prokaryotic community in the deeper layer during the stratified period, the frequency of the small virus-infected prokaryotes (<0.02 μm^3^: very left bar in each plot) was reduced slightly and the frequency of the larger virus-infected prokaryotes (0.02–0.1 μm^3^) increased compared to that of the total prokaryotic community. This suggests that viruses tend to infect relatively larger prokaryotes in the prokaryotic community. This is consistent with the findings of a previous study, wherein size-specific viral infection was observed in Lake Pluβsee (Germany; Weinbauer and Höfle, 1998b) and probably because the estimated contact rate of large cells was higher than that of smaller cells (Murray and Jackson, 1992; Weinbauer and Höfle, 1998b). These data suggest that larger prokaryotes are susceptible to be infected by viruses and mortality due to viral infection could be reduced in smaller prokaryotes. In addition, when the proportion of larger prokaryotes increases, these prokaryotes can maintain a larger number of intracellular viral particles than smaller prokaryotes. In the deeper layer, during the stratified period, the number of intracellular viral particles tended to increase with an increase in the cell volume of infected prokaryotes (Figure 7). This is also supported by the results of a previous study conducted in Lake Pluβsee (Germany; Weinbauer and Peduzzi, 1994). Thus, in environments with low activity of prokaryotes or low contact rates, larger prokaryotes might be important to increase the estimated contact rate and the number of intracellular viral particles, resulting in productive viral infection and maintenance of viral abundance.

### Virus-Lysed DOM Could Contribute to the Labile DOM Pool

Virus-lysed DOM are regarded as labile for prokaryotic utilization (Middelboe and Jørgensen, 2006) and can be quickly utilized by other heterotrophic prokaryotes (Zheng et al., 2020). Here, we estimated the abundance – using the estimated volume of infected cells – and the contribution of lysed DOM to the DOM pool in Lake Biwa. Virus-lysed DOM contributed up to 0.89 μg C L^−1^ d^−1^, 0.21 μg N L^−1^ d^−1^, and 0.035 μg P L^−1^ d^−1^ in the surface layer and 0.057 μg C L^−1^ d^−1^, 0.013 μg N L^−1^ d^−1^, and 0.0023 μg P L^−1^ d^−1^ in the deeper layer (Table 3).

**Table 3:** Mean ± SD (median and range) for physical, chemical, and biological parameters during the research period (from August 2016 through January 2018).

Parameters (units)	Stratified		De-stratified
	0.5 m	60 m	
Water temperature (°C)	20.1 ± 6.4 (21.1, 10.6–28.9)	8.3 ± 0.4 (8.3, 7.8–8.9)	8.2 ± 0.4 (8.3, 7.7–8.7)
Dissolved oxygen (mg L^−1^)	9.4 ± 1.1 (9.3, 7.8–11.4)	8.2 ± 1.4 (7.9, 6–10.7)	10.7 ± 0.5 (10.6, 10.1–11.5)
Prokaryotic abundance (10^9^ cells L^−1^)	2.0 ± 0.8 (2.1, 0.8–3.5)	0.7 ± 0.2 (0.8, 0.3–0.9)	1.4 ± 0.6 (1.0, 0.8–2.3)
Viral abundance (10^10^ L^−1^)	4.9 ± 2.4 (4.5, 1.9–11)	1.9 ± 0.8 (1.7, 1.0–4.1)	3.8 ± 0.8 (3.5, 2.6–5.5)
Virus-to-prokaryotes ratio	26.4 ± 16.8 (19.2, 8.3–58.0)	28.8 ± 11.6 (27.4, 13.4–51.6)	24.0 ± 14.1 (18.5, 11.2–54.0)
Prokaryotic production (10^9^ cells L^−1^ d^−1^)	0.31 ± 0.20 (0.27, 0.07–6.7)	0.023 ± 0.010 (0.019, 0.007–0.044)	0.034 ± 0.007 (0.036, 0.023–0.040)
Frequency of visibly infected cells (%)	2.0 ± 0.9 (1.9, 0.9–3.8)	2.0 ± 0.6 (2.0, 1.1–3.0)	1.7 ± 0.5 (1.7, 1.1–2.4)
Fraction of prokaryotic mortality due to viral lysis (%)	25 ± 17 (19, 5.9–67)	22 ± 9.8 (21, 7.9–43)	17 ± 7.3 (17, 8.0–29)
Carbon release (μg C L^−1^ d^−1^)	0.31 ± 0.33 (0.12, 0.018–0.89)	0.033 ± 0.013 (0.036, 0.013–0.057)	0.011 ± 0.005 (0.01, 0.006–0.019)
Nitrogen release (μg N L^−1^ d^−1^)	0.07 ± 0.077 (0.029, 0.004–0.21)	0.0078 ± 0.0032 (0.0085, 0.003–0.013)	0.0027 ± 0.0012 (0.0025, 0.0014–0.0044)
Phosphorus release (μg P L^−1^ d^−1^)	0.012 ± 0.013 (0.005, 0.001–0.035)	0.0013 ± 0.0005 (0.0014, 0.0005–0.0023)	0.0005 ± 0.0002 (0.0004, 0.0002–0.0008)
Concentration of bulk dissolved organic carbon (mg C L^−1^)	1.25 ± 0.07 (1.26, 1.08–1.35)	1.03 ± 0.06 (1.02, 0.94–1.15)	1.11 ± 0.07 (1.07, 1.06–1.22)
Number of intracellular viral particles (viruses cell^−1^)	12 ± 11 (9, 3–100)	33 ± 29 (23, 3–124)	21 ± 23 (12, 3–132)
Infected cell length (μm)	0.84 ± 0.44 (0.74, 0.27–3.33)	1.60 ± 0.85 (1.52, 0.39–3.59)	0.87 ± 0.63 (0.63, 0.30–4.34)
Infected cell volume (μm^3^)	0.044 ± 0.058 (0.031, 0.0018–0.76)	0.096 ± 0.10 (0.065, 0.010–0.54)	0.050 ± 0.12 (0.024, 0.0061–1.26)
Intracellular viral capsid diameter (nm)	72 ± 26 (64, 24–161)	56 ± 25 (48, 29–139)	56 ± 22 (54, 23–123)
Estimated contact rate (contacts cell^−1^ d^−1^)	102 ± 65 (78, 27–273)	38 ± 11 (37, 25–70)	42 ± 12 (40, 28–67)

The de-stratified periods were January–March 2017, and January 2018, at which time lake turnover had occurred and the surface and deeper layer mixed. Dataset of water temperature and concentration of dissolved oxygen were obtained from Shiga Prefecture (Shiga-Prefecture, 2017, 2018).

In Lake Biwa, bulk DOC concentrations ranged from 1.08 to 1.35 mg C L^−1^ in the surface layer and from 0.94 to 1.15 mg C L^−1^ in the deeper layer during the stratified period (Supplementary Table 6). Additionally, the refractory DOC concentration was estimated to be 0.98 mg C L^−1^, and the concentration of semi-labile DOC was estimated to be 0.20 and 0.078 mg C L^−1^ in the surface and deeper layers, respectively (Maki et al., 2010). Assuming that the total DOC consisted of refractory, semi-labile, and labile DOC, the sum of refractory and semi-labile DOC concentrations accounted for 94.9 ± 4.6% (mean ± SD) and 101.2 ± 5.0% (mean ± SD) of bulk DOC concentration in the surface and deeper layer, respectively (Supplementary Table 6). This suggests that in the deeper layer, labile DOC, which can be estimated as the subtraction of refractory and semi-labile DOC concentrations from bulk DOC concentration, is consumed almost entirely and depleted. This is probably because in the deeper layer, net primary production is ≤0 g C m^−3^ y^−1^, suggesting that new organic matter production is limited and the deeper layer is a decomposition layer (Kishimoto et al., 2015). In this case, lysed DOM could be an important source of organic substrates for heterotrophic prokaryotes in the deeper layer. Moreover, large prokaryotes being lysed *via* viral infection could contribute to the labile DOM pool.

## Conclusion

This study shows seasonal variations in the viral infection rates and cell sizes of infected prokaryotes in the surface and deeper layers of Lake Biwa. We propose the mechanisms underlying the maintenance of viral abundance in the two water layers. In the surface layer, it is speculated that the high viral infection rate leads to viral abundance because of the high prokaryote activity during the middle of the stratified period, whereas in the deeper layer, it is speculated that viral abundance might be supported by the larger number of intracellular viral particles released from large prokaryotes at the end of the stratified period. Moreover, large prokaryotes could serve as important sources of organic substrates *via* viral lysis in the deeper layer, where labile DOM is depleted. Although further research is needed to unravel the source of prokaryotic mortality at the species level and the fate of virus-induced DOM, our results highlight the importance of prokaryote lysis in a deep freshwater lake.

## Data Availability Statement

The raw data supporting the conclusions of this article will be made available by the authors, without undue reservation.

## Author Contributions

SS contributed to the research design, the analysis, results, discussion, and manuscript preparation. YS contributed to the discussion, manuscript revision, and overall support for this study. All authors contributed to the article and approved the submitted version.

### Conflict of Interest

The authors declare that the research was conducted in the absence of any commercial or financial relationships that could be construed as a potential conflict of interest.
