# Supplementary material for: Seasonal Variation in Viral Infection Rates and Cell Sizes of Infected Prokaryotes in a Large and Deep Freshwater Lake (Lake Biwa, Japan)
Source: Front Microbiol. 2021 May 11;12:624980. doi: 10.3389/fmicb.2021.624980 (PMC8144228; doi:10.3389/fmicb.2021.624980)
Supplement: Supplementary file 1 [file Data_Sheet_1.pdf]

Supplementary Material

1 Supplementary Tables

Supplementary Table 1. Statistical analyses.

	Sample	Comparison	Values used for comparison	Sample size	Statistics
**Viral abundance**	Each month	Between the surface and deeper layer	Mean	10	Welch's *t*-test
**Viral abundance**	Season (stratified and de-stratified period)	Between the surface and deeper layer	Mean	14 during the stratified period and 4 during the de-stratified period	Student's *t*-test
**FVIC**	Each month	Each month	Mean	3	Student's *t*-test
**FVIC**	Season (stratified and de-stratified period)	Between the surface and deeper layer	Mean	14 during the stratified period and 4 during the de-stratified period	Student's *t*-test
**Cell volume distribution**	Season (de-stratified, and beginning, middle, and end of the stratified period)	Between the surface and deeper layer	Median	34–653	Mann-Whitney *U* test
**Cell volume distribution**	Season (de-stratified, and beginning, middle, and end of the stratified period)	Between the total prokaryotic community and virus-infected prokaryotes	Median	34–653	Mann-Whitney *U* test

FVIC, frequency of visibly infected cells

Supplementary Table 2. Statistical significance in the cell volume differences between the surface and deeper layers. The values in each cell indicate the p-value. Original data are shown in Figures 1B and 1D.

		Surface vs. Deeper layers	
	Date	Viral abundance	FVIC
2016	August	(4.9×10^{-9})***	(0.0070)**
	September	(1.3×10^{-5})***	(0.17) NS
	October	(7.5×10^{-7})***	(0.49) NS
	November	(3.6×10^{-8})***	(0.032)*
	December	(1.3×10^{-8})***	(0.0063)**
2017	January	(0.43) NS	(0.49) NS
	February	(0.0025)**	(0.54) NS
	March	(0.0051)**	(0.097) NS
	April	(1.4×10^{-7})***	(0.073) NS
	May	(5.6×10^{-9})***	(0.17) NS
	June	(0.0071)**	(0.78) NS
	July	(1.5×10^{-4})***	(0.012)*
	August	(8.1×10^{-11})***	(0.0089)**
	September	(3.5×10^{-7})***	(0.0040)**
	October	(8.7×10^{-8})***	(0.42) NS
	November	(1.3×10^{-6})***	(0.0047)**
	December	(2.2×10^{-5})***	(0.017)*
2018	January	(3.2×10^{-6})***	(0.41) NS
	De-stratified	(0.57) NS	(0.79) NS
	Stratified	(0.0033)**	(0.87) NS

*$p < 0.05$, **$p < 0.01$, *** $p < 0.001$, NS No significant difference
FVIC, frequency of visibly infected cells

Supplementary Table 3.

U-values from the Mann-Whitney U test for comparing the cell volume between the surface and deeper layers. Related results are shown in Table 1 and Figure 3.

	Difference between the surface and deeper layers			
	De-stratified	Stratified -beginning -	Stratified -middle-	Stratified -end-
Total prokaryotic community	103,788	45,469	73,277	100,212
Infected prokaryotes	2,126	896	1,172	621

Supplementary Table 4.

U-values from the Mann-Whitney U test for comparing the cell volume between the total prokaryotic community and infected prokaryotes. Original data are shown in Figure 7. Related results are shown in Table 2 and Figure 3.

	Difference between total prokaryotic community and virus-infected prokaryotes			
	De-stratified	Stratified -beginning -	Stratified -middle-	Stratified -end-
0.5 m	14,649	11,507	51,344	27,196
60 m	18,008	7,051	9,118	18,137

Supplementary Table 5.

Statistical significance of differences in the values of cell volume of infected cells and the number of intracellular viral particles between each layer. Original data are shown in Figures 6 and 7.

		At 0.5 m vs. 60 m during the stratified period	At 0.5 m during the stratified period vs. both layers during the de-stratified period	At 60 m during the stratified period vs. both layers during the de-stratified period
Cell volume of infected cells	p-value	(3.0×10^{-14})***	(0.0021)**	(7.3×10^{-14})***
	U-value	8,760	21,243	12,257
Number of intracellular viral particles	p-value	(9.5×10^{-13})***	(7.7×10^{-5})***	(0.00088)***
	U-value	8382	13,532	9,162
Viral capsid diameter inside infected cells	p-value	(7.2×10^{-9})***	(7.2×10^{-9})***	(0.87)NS
	U-value	20,373	23,735	6,947

*$p < 0.05$, **$p < 0.01$, *** $p < 0.001$, NS No significant difference

Supplementary Table 6.

Summary of the bulk, refractory, and semi-labile DOC concentrations (mg C L^{-1}) during the stratified period. Refractory and semi-labile DOC concentrations were obtained from (Maki et al., 2010). The numbers indicate mean ± SD.

Depth	Bulk DOC (a)	Refractory DOC (b)	Semi-labile DOC (c)	b + c	%, (b + c)/a
0.5 m	1.25 ± 0.07<br>1.08–1.35	0.98	0.20	1.18	94.9 ± 4.6<br>87–101
60 m	1.03 ± 0.06<br>0.94–1.15	0.98	0.08	1.06	101.2 ± 5.0<br>92–108
